# Community-based physical activity interventions for prevention and management of non-communicable diseases in high-income countries vs. low- and middle-income countries: a narrative review

**DOI:** 10.3389/fspor.2026.1833314

**Published:** 2026-10-30

**Authors:** Mandisa Jewel Simamane, Musa Lewis Mathunjwa

**Affiliations:** 1 University of the Witwatersrand, Johannesburg, South Africa; 2 Department of Human Movement Science, University of Zululand, KwaDlangezwa, South Africa

**Keywords:** community-based interventions, high-income countries, low- and middle-income countries, non-communicable diseases, physical activity

## Abstract

**Background:**

Non-communicable diseases (NCDs) represent a major global health challenge, accounting for approximately 74% of global deaths and nearly 43 million deaths annually in 2025. Cardiovascular disease mortality has generally declined in many high-income countries, while many low- and middle-income countries (LMICs) continue to experience a substantial burden. Physical inactivity remains an important behavioural risk factor contributing to this burden.

**Objective:**

This narrative review evaluates the effectiveness, feasibility, and sustainability of community-based physical activity (PA) interventions, comparing high-income countries (HICs) and LMICs to identify context-sensitive strategies and transferable lessons for global NCD prevention.

**Methods:**

A structured literature search and narrative synthesis, guided by Popay et al., were conducted on English-language research and relevant institutional reports published from January 2006 to 18 March 2026. Searches covered PubMed/MEDLINE, Scopus, Web of Science, and the Cochrane Library. The narrative synthesis drew on 46 cited sources; eight representative evidence sources are summarised in Table 3. The review was designed as a narrative review rather than a systematic review, and therefore no PRISMA-style study-selection flow was undertaken.

**Results:**

The synthesis indicates that community-based PA interventions are implemented through combinations of infrastructure, professional or peer delivery, behavioural support, and community adaptation. The representative evidence includes quantitative findings from controlled studies and meta-analyses as well as implementation and contextual evidence. Effects are shaped by implementation capacity, contextual barriers, and the fit between programme design and local needs; the available LMIC evidence is less extensive than the broader HIC evidence base.

**Conclusion:**

Community-based PA interventions can support NCD prevention and management across diverse settings, but implementation should be adapted to available resources, health-system capacity, cultural context, safety, and community preferences. The Layered Socio-Ecological Comparison Framework is presented as an author-developed synthesis tool informed by established ecological and health-promotion frameworks, rather than as a validated standalone theory.

## 1 Introduction

Non-communicable diseases (NCDs), primarily arising from behavioural, environmental, and metabolic risk factors, pose significant global health challenges (2, 3). In 2025, NCDs were responsible for approximately 74% of global deaths, equating to around 43 million deaths annually (2, 3). Major NCDs include cardiovascular diseases (CVDs), cancer, chronic respiratory conditions, hypertension, and diabetes mellitus, with CVDs alone contributing an estimated 523 million cases and 18.6 million deaths globally (4, 5). Key behavioural risk factors include unhealthy diets, physical inactivity, and substance use, which increase the risk of NCDs such as type 2 diabetes and hypertension (6–8). NCDs also carry substantial socioeconomic costs, including healthcare expenditure, reduced productivity, and premature mortality (9).

Between 1990 and 2019, age-standardised CVD deaths declined in high-income nations (HICs) and some middle-income nations, whereas many LMICs experienced substantial increases in CVD burden (10, 11). Differences in CVD burden across regions are influenced by demographic change, socioeconomic shifts, epidemiological transitions, behavioural risk acquisition, globalisation, and industrialisation (10–12). Population-level lifestyle interventions, particularly those promoting PA, have been implemented in both HICs and LMICs and can improve PA levels and reduce cardiovascular risk (13–15).

Regular PA is a cornerstone in the prevention of lifestyle-related diseases, including CVD and type 2 diabetes, by improving cardiovascular health, fitness, and related health outcomes (2, 16, 17). Community-based interventions and multifactorial strategies targeting PA and other behavioural risks have demonstrated benefits for activity levels and cardiovascular risk (18–21). Effective interventions require attention to target behaviours, motivational and social factors, and community-specific needs, and may include structured programmes, supportive policies, dance, games, and sport (22–25). While modifying and sustaining PA habits remains challenging (26), recommended targets such as 150 min of moderate-intensity or 75 min of vigorous-intensity aerobic activity per week provide substantial health benefits (27). Therefore, the aim of this narrative review is to explore effectiveness, feasibility, sustainability, and contextual moderators influencing impact, with the aim of identifying transferable lessons and context-sensitive strategies to inform global NCD prevention efforts.

## 2 Theoretical framework

The Layered Socio-Ecological Comparison Framework is presented in this review as an author-developed conceptual synthesis tool for organising evidence on community-based PA interventions across high-income countries (HICs) and low- and middle-income countries (LMICs). The terminology HICs and LMICs is used consistently throughout the manuscript; these economic categories are treated as contextual descriptors rather than homogeneous or deterministic groupings. The framework is not presented as a newly validated theory. Instead, its four levels draw on established ecological and health-promotion perspectives, particularly the ecological model of health promotion, the Ottawa Charter for Health Promotion, and the PRECEDE-PROCEED approach (28–30).

The framework (Figure 1) integrates these established perspectives with the comparative focus of the present review. Level 1 addresses structural context; Level 2 addresses implementation models; Level 3 addresses behavioural mechanisms and contextual modifiers; and Level 4 addresses clinical, behavioural, and system-level outcomes. The framework is therefore used as an organising device for synthesis and comparison, not as a validated measurement instrument or causal model.

**Figure 1:**
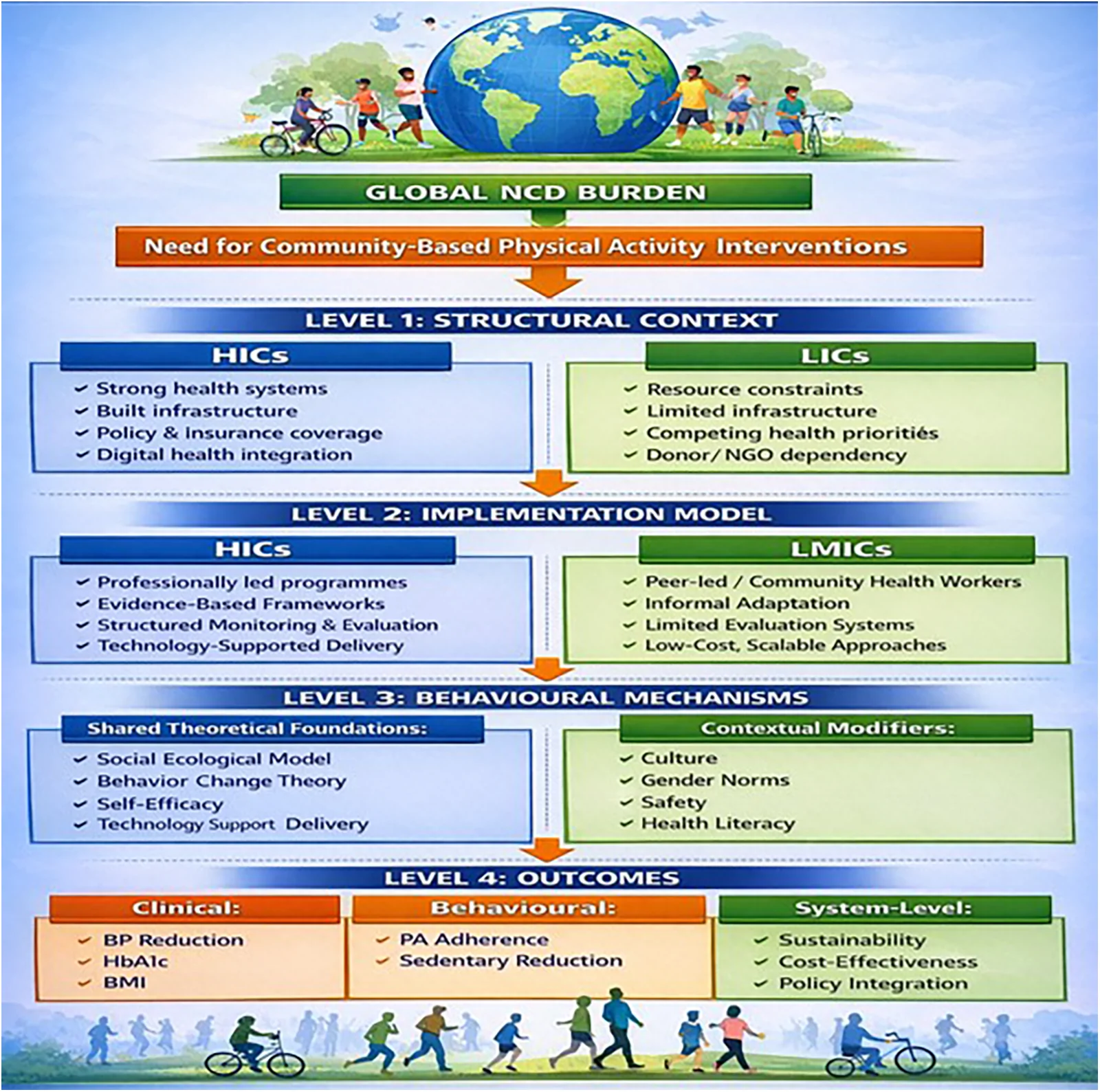
Layered socio-ecological comparison framework. HICs, high-income countries; LMICs, low- and middle-income countries; PA, physical activity; NCDs, non-communicable diseases. The framework is an author-developed conceptual synthesis tool for organising evidence and is not a validated measurement instrument or causal model.

At the foundational stage, Level 1: Structural Context examines the baseline infrastructure available for health promotion. HICs may have stronger health-system capacity, established built infrastructure, digital health integration, and policy or insurance mechanisms, whereas LMIC settings may face resource constraints, competing health priorities, limited infrastructure, and greater dependence on community or external support. These structural conditions can shape Level 2: Implementation Model, where interventions may range from professionally led and technology-supported programmes to peer-led and community-health-worker approaches. The evidence is heterogeneous, and these patterns are interpreted as context-dependent tendencies rather than universal characteristics of all HICs or LMICs.

The framework then transitions to Level 3: Behavioural Mechanisms, which identifies psychological and social drivers of change. Established perspectives such as the Social Ecological Model, behaviour-change approaches, and self-efficacy provide the theoretical foundation, while contextual modifiers determine how these mechanisms operate in particular communities. Culture, gender norms, safety, health literacy, social support, and access to facilities may influence participation and maintenance. Level 4: Outcomes synthesises clinical indicators such as blood pressure, glycaemic measures and BMI; behavioural indicators such as physical activity adherence and sedentary time; and system-level indicators such as feasibility, sustainability, cost, and policy integration. The framework thus provides a transparent structure for comparing evidence while acknowledging that the strength and direction of relationships vary across settings.

Table 1 provides a high-level conceptual comparison of infrastructure, workforce, technology, funding, and community adaptation factors. It summarises contextual tendencies and is not intended to imply that all HICs or LMICs share the same characteristics.

**Table 1:** Structural features relevant to community-based physical activity delivery in HICs and LMICs.

Feature	High-income countries (HICs)	Low- and middle-income countries (LMICs)
Infrastructure	Established facilities, health-system capacity and potential digital integration	Variable infrastructure, resource constraints and competing priorities
Leadership/workforce	Professional or multidisciplinary delivery may be available	Peer, community and community-health-worker delivery may be used
Technology	Digital or mobile tools can supplement delivery	Access and digital literacy may constrain technology-dependent models
Funding/sustainability	Potential integration with established services or funding mechanisms	External or short-term funding may play a larger role in some settings
Community adaptation	Programmes may still require adaptation for equity and adherence	Local adaptation may be essential to feasibility and acceptability

## 3 Materials and methods

The methodology for this review was designed as a narrative review using a structured literature search and narrative synthesis. This approach was selected because the literature includes heterogeneous intervention designs, populations, outcomes, implementation models, and contextual descriptions that are not readily reducible to a single quantitative effect estimate. The review follows the principles of transparent narrative synthesis described by Popay et al. (1), while avoiding claims that it constitutes a systematic review.

### 3.1 Search strategy and information sources

A structured search was conducted across PubMed/MEDLINE, Scopus, Web of Science, and the Cochrane Library. The search combined database-specific subject headings and keywords across three clusters: (1) community setting (e.g., “community-based”, “neighbourhood-led”, “grassroots”); (2) physical activity (e.g., “physical activity”, “exercise”, “sedentary behaviour”); and (3) NCD outcomes or risk (e.g., “non-communicable diseases”, “cardiovascular risk”, “type 2 diabetes”). An example combined search structure was: (“community-based” OR “neighbourhood-led” OR grassroots) AND (“physical activity” OR exercise OR “sedentary behaviour”) AND (“non-communicable diseases” OR “cardiovascular risk” OR “type 2 diabetes”). Database-specific subject headings and syntax were adapted to each database. Searches were limited to English-language peer-reviewed literature and relevant institutional reports published from January 2006 through 18 March 2026.

### 3.2 Eligibility criteria and literature selection

Studies were considered eligible when they: (1) described a community-based physical activity, exercise, or physical-activity-promotion intervention; (2) involved community-dwelling participants; (3) addressed prevention or management of NCDs or major NCD risk factors; (4) provided information on intervention delivery, context, feasibility, implementation, or outcomes; and (5) were published in English between January 2006 and 18 March 2026. Studies were excluded when they focused exclusively on pharmacological treatment, elite athletic performance, inpatient or highly specialised clinical rehabilitation, or physical activity without a relevant NCD-prevention or management component. The economic-context comparison was based on the classification reported by the source study; HICs and LMICs were compared descriptively rather than treated as homogeneous groups.

Table 2 defines the population, intervention, comparator, outcomes, study characteristics, date/language limits, and exclusion criteria used to determine whether a source was relevant to the narrative synthesis.

**Table 2:** Structured eligibility criteria (PICO(S)) for the narrative review.

PICO(S) element	Eligibility criterion
Population	Community-dwelling adults, adolescents, or children in HICs or LMICs, including populations at risk of or living with NCDs or major NCD risk factors.
Intervention	Community-based physical activity, exercise, physical-activity-promotion, or behaviour-support interventions delivered in community, school, neighbourhood, workplace, or comparable non-inpatient settings.
Comparator	Usual care, no intervention, baseline/pre-intervention status, alternative physical activity intervention, or contextual comparison between HIC and LMIC settings, where reported.
Outcomes	Physical activity, fitness, anthropometric or cardiometabolic indicators, NCD risk/management, adherence, feasibility, acceptability, reach, implementation, sustainability, cost, or policy/system outcomes.
Study design	Systematic reviews, meta-analyses, randomised or non-randomised intervention studies, mixed-methods/implementation studies, qualitative or narrative evidence, and authoritative institutional reports where directly relevant.
Limits	English-language literature published January 2006–18 March 2026. No post-submission publication was included in the formal search or evidence base.
Exclusion	Pharmacological-only treatment, elite athletic performance, inpatient or highly specialised clinical rehabilitation, and physical activity studies without a relevant NCD-prevention or management component.

### 3.3 Narrative synthesis framework

The narrative synthesis was guided by Popay et al. (1) through four linked activities: developing an initial theory of how interventions may work; producing a preliminary synthesis; exploring relationships within and between studies; and considering the robustness and limitations of the resulting synthesis. Findings were organised around structural context, implementation model, behavioural mechanisms/contextual modifiers, and outcomes. The aim was to identify patterns and differences without implying that all included studies were directly comparable.

### 3.4 Data extraction and evidence appraisal

Data synthesis was iterative and comparative. For representative sources, information was extracted under the following fields: author/year, country or setting, income context where identifiable, study design, population/sample, intervention and delivery model, reported outcomes, effect direction and effect size where available, contextual factors, and implementation considerations. Priority was given to systematic reviews, meta-analyses, peer-reviewed empirical studies, and authoritative health reports when making broad claims. Individual studies and contextual sources were used to illustrate mechanisms or setting-specific experiences. Because this was a narrative review, no formal Cochrane, CASP, or MMAT risk-of-bias score was assigned to every source; instead, each source was appraised qualitatively according to study design, directness to the review question, consistency of findings, sample or evidence-base size, and relevance to the economic context. The representative evidence table makes this appraisal explicit, while the strength of conclusions is moderated where evidence is preliminary, contextual, or indirect.

The synthesis was organised using the four levels of the framework while retaining the broader ecological perspective of individual, interpersonal, community, organisational, and policy influences. Contradictory or context-dependent findings were retained rather than resolved into a single universal model. For example, technology-supported delivery may improve reach or monitoring in some HIC settings, whereas low-cost group-based or peer-led approaches may be more feasible where digital access or professional resources are constrained. This approach was used to identify transferable principles while avoiding assumptions that an intervention can be transferred unchanged between economic contexts.

### 3.5 Study-selection transparency

Because the review is explicitly classified as a narrative review, the authors did not conduct a systematic-review screening process and therefore did not generate a PRISMA flow diagram or a formal count of records identified, screened, excluded, and included. To improve transparency without implying an exhaustive systematic search, the manuscript now reports that 46 cited sources informed the narrative synthesis and that eight representative evidence sources are presented in Table 3. The selection was relevance-based and the synthesis retained differences in study design, population, setting, and outcome rather than treating all sources as directly comparable.

**Table 3:** Representative evidence informing the narrative synthesis.

Study/source	Setting, income context and design	Population/intervention	Reported outcomes: direction and size	Contribution to synthesis/evidence appraisal
Hassen et al. (21)	Multinational; mixed HIC/LMIC evidence; systematic review and meta-analysis of 64 controlled studies (44 randomised, 20 non-randomised)	Community-based CVD-prevention interventions; 98,919 participants	Improved recommended PA at 12 months (OR 1.62, 95% CI 1.25–2.11) and 18–24 months (OR 1.46, 95% CI 1.12–1.91); METs SMD 0.28, MVPA SMD 0.34, steps SMD 0.32, sitting time SMD −0.25. LMIC evidence was limited.	Strong quantitative evidence for PA outcomes; relevant LMIC evidence remained limited, so findings should not be generalised as equally representative of all economic contexts.
Mathunjwa et al. (31)	South Africa; LMIC context; quasi-experimental community programme	132 recruited; 125 assigned to intervention/control groups (60 intervention, 65 control); 10-week Tae-Bo, 45–60 min, three times weekly	Significant reductions in body weight (*p* = 0.043), BMI (*p* = 0.037), waist circumference (*p* = 0.031) and hip circumference (*p* = 0.040); improvements also reported for flexibility, balance, muscular fitness and cardiorespiratory endurance.	Direct low-resource community intervention evidence; supports contextual adaptation and feasibility while remaining specific to one rural South African setting.
Lee et al. (32)	Hong Kong; HIC context; 2-arm pilot feasibility RCT	38 community-dwelling older adults randomised 19/19; four weekly workshops plus mobile app and outdoor exercise facilities	34/38 completed (89%); high workshop attendance; both groups increased PA immediately, but only the intervention group maintained the increase at 3 months; favourable changes in mental well-being and self-efficacy were observed.	Shows feasibility of technology-supported delivery in an HIC setting and the importance of adherence and sustained engagement.
Whooten et al. (24)	Massachusetts, USA; HIC context; mixed-methods implementation evaluation	188/230 places filled; 128 children provided study data; before-school PA programme in three schools	Mean 16.3 (SD 9.3) min MVPA per session; median attendance 90%; no change in BMI z-score or self-reported quality of life; school-level adaptations were documented.	Implementation evidence showing that reach, adaptation and delivery conditions matter even in a high-income setting.
Ma et al. (33)	Systematic review and meta-analysis; 10 eligible RCTs; mixed settings	2,349 participants across habit-formation interventions targeting PA	PA habit strength improved vs. control (SMD 0.31, 95% CI 0.14–0.48, *p* < 0.001).	Quantitative behavioural-maintenance evidence supporting the habit and adherence component of the framework.
Shandu et al. (34)	South Africa; LMIC context; randomised exercise intervention	60 physically inactive college-aged male smokers; 48 analysed; 8-week HIIT or continuous aerobic cycling, three sessions weekly	HIIT improved selected lung-function outcomes; both exercise groups improved recovery heart rate and cardiorespiratory-related outcomes.	Adds intervention-level evidence that exercise prescription can be tailored to specific risk groups; transferability is limited by the specific population and non-community setting.
Mathunjwa et al. (35)	South Africa; LMIC context; narrative review	Community-centred approaches for people living with T2DM and HIV	Synthesised culturally relevant, scalable, peer-support and community-based approaches for healthy living in resource-constrained settings.	Contextual evidence supporting community-health and implementation considerations; qualitative/narrative rather than pooled effect evidence.
Mathunjwa et al. (36)	African communities; predominantly LMIC contexts; public-health perspective/narrative evidence	Indigenous games and culturally familiar PA for NCD prevention	Identified indigenous games as potentially accessible and culturally resonant forms of PA for African communities.	Supports cultural adaptation and community ownership as contextual modifiers; does not provide a pooled intervention effect estimate.

## 4 Results

### 4.1 Evidence base and characteristics of representative studies

The final reference list contains 46 cited sources comprising empirical studies, systematic reviews, methodological guidance, policy or health reports, and conceptual sources. Because this manuscript is a narrative review rather than a systematic review, a formal PRISMA screening count was not generated. The synthesis therefore reports the number of cited sources and provides eight representative evidence sources in Table 3, selected to demonstrate quantitative intervention effects, implementation findings, behavioural evidence, and contextual evidence across HIC and LMIC settings.

The representative evidence underlying these observations is detailed in Table 3, including study design, participant characteristics, intervention format, outcomes, and qualitative evidence appraisal.

The representative evidence is heterogeneous but sufficiently detailed to identify recurring patterns. Hassen et al. (21) provides quantitative evidence from 64 controlled studies involving 98,919 participants. Mathunjwa et al. (31) provides a low-resource rural South African intervention with 125 participants assigned to intervention/control groups, while Lee et al. (32) provides a 38-participant high-income pilot and Whooten et al. (24) provides an implementation-focused evaluation with 128 study participants. Ma et al. (33) adds quantitative evidence on behavioural maintenance from 10 studies involving 2,349 participants. These sources illustrate the importance of reporting both intervention effects and implementation conditions.

Table 3 summarises eight representative evidence sources used to support the narrative synthesis. It reports the setting and economic context where identifiable, design, sample/population, intervention or evidence type, reported outcomes including effect direction and size where available, and the contribution and limitations of each source to the synthesis. The table is illustrative rather than an exhaustive systematic-review inventory.

### 4.2 Comparative structural environments

The evidence indicates that structural capacity influences the feasible design and delivery of community-based physical activity interventions, but economic classification alone does not determine intervention form. In HIC settings, community programmes may make use of established facilities, professional staff, digital technologies, and structured implementation systems. In LMIC settings, interventions may rely more heavily on community participation, peer delivery, existing community spaces, or low-cost formats. These should be understood as recurring patterns in the reviewed literature rather than fixed characteristics of every country.

### 4.3 Diversity in implementation strategies

Implementation strategies varied substantially across studies. Hassen et al. (21), in a systematic review and meta-analysis of 64 controlled studies involving 98,919 participants, found that community-based cardiovascular disease-prevention interventions improved attainment of recommended physical activity levels at 12 months (OR: 1.62, 95% CI: 1.25–2.11) and at 18–24 months (OR: 1.46, 95% CI: 1.12–1.91). The review also reported improvements in metabolic equivalents, moderate-to-vigorous physical activity, steps per day, and sitting time, while noting that evidence from lower-income settings remained limited. This finding supports the feasibility of community interventions while also demonstrating that the evidence base is uneven across economic contexts.

Table 4 compares structural and implementation characteristics across HIC and LMIC settings using representative sources. It is intended as a contextual comparison rather than a universal description of either group.

**Table 4:** Structural and implementation comparison across HIC and LMIC settings.

Feature	High-Income Countries (HICs)	Low- and middle-income countries (LMICs)	Interpretation	Representative references
Infrastructure	Established facilities, health-system capacity and potential digital integration	Variable infrastructure, resource constraints and competing priorities	Infrastructure shapes feasibility, reach and maintenance	(15, 22)
Leadership/workforce	Professional or multidisciplinary delivery may be available	Peer, community and community-health-worker delivery may be used	Workforce availability influences reach and scalability	(31, 37)
Technology	Digital or mobile tools can supplement delivery	Access and digital literacy may constrain technology-dependent models	Technology should match access and implementation capacity	(32)
Funding/sustainability	Potential integration with established services or funding mechanisms	External or short-term funding may play a larger role in some settings	Long-term sustainability depends on institutional integration	(14, 22)
Community adaptation	Programmes may still require adaptation for equity and adherence	Local adaptation may be essential to feasibility and acceptability	Economic classification does not remove the need for contextual tailoring	(24, 31)
Leadership/workforce	Professional or multidisciplinary delivery may be available	Peer, community and community-health-worker delivery may be used	Workforce availability influences reach and scalability	(21, 37)
Technology	Digital or mobile tools can supplement delivery	Access and digital literacy may constrain technology-dependent models	Technology should match access and implementation capacity	(32)
Funding/sustainability	Potential integration with established services or funding mechanisms	External or short-term funding may play a larger role in some settings	Long-term sustainability depends on institutional integration	(14, 22)
Community adaptation	Programmes may still require adaptation for equity and adherence	Local adaptation may be essential to feasibility and acceptability	Economic classification does not remove the need for contextual tailoring	(24, 31)

Table 5 identifies contextual modifiers that can influence participation, acceptability, reach, adherence, and sustainability across economic settings.

**Table 5:** Contextual modifiers affecting intervention delivery and maintenance (level 3).

Modifier	Observed/possible impact on intervention	Contextual relevance	Illustrative evidence
Culture and gender	Influences social acceptability, access, participation and preferred delivery settings	Requires culturally responsive and gender-sensitive design	(38, 39)
Safety	Crime, traffic and environmental safety may affect outdoor participation	Safe spaces can determine whether programmes are practically accessible	(39)
Health literacy	Affects understanding of risk, intervention instructions and self-management	Communication and education should be adapted to participants' needs	(40)
Digital access and literacy	Can facilitate monitoring and support but may exclude people without access or skills	Technology should be treated as an enabling component rather than a prerequisite	(32)
Social support and community ownership	Can reinforce participation, adherence and maintenance	Peer and community structures may support sustainability when appropriately resourced	(31, 37)

Table 6 organises outcomes into clinical/anthropometric, behavioural, implementation, and system-level domains to clarify how intervention effects were interpreted.

**Table 6:** Categorisation of intervention outcomes (Level 4).

Category	Primary indicators	Interpretation	Representative evidence
Clinical/anthropometric	Body weight, BMI, waist/hip circumference, blood pressure and other NCD risk indicators	Indicates physiological response but varies by intervention and population	(21, 31)
Behavioural	MVPA, total PA, steps, sedentary time, adherence and habit strength	Captures behaviour change and maintenance	(21, 33)
Implementation	Reach, attendance, feasibility, acceptability, adaptation and fidelity	Explains why an intervention may succeed or fail in a specific setting	(24, 32)
System-level	Sustainability, cost, workforce capacity and policy integration	Determines whether benefits can be maintained beyond the study period	(14, 22)

## 5 Discussion

The synthesis indicates that differences between HIC and LMIC settings are better understood as differences in enabling conditions, implementation capacity, and contextual constraints than as simple differences in intervention effectiveness. Evidence from controlled studies supports the potential value of community-based interventions, but the available literature is uneven by geography, study design, population, and outcome. The comparison therefore identifies context-sensitive implementation principles rather than a single model that should be applied across countries.

### 5.1 Effectiveness and structural capacity

Evidence from the Hassen et al. (21) meta-analysis provides quantitative support within the current evidence base: community-based cardiovascular prevention interventions improved recommended PA attainment and several activity-related outcomes over follow-up. The review included 44 randomised and 20 non-randomised controlled studies involving 98,919 participants and reported improvements in recommended PA attainment, METs, MVPA, steps per day, and sitting time. Importantly, relevant studies in lower-income settings were limited. The Mathunjwa et al. (31) study adds context-specific evidence from a low-resource rural South African setting, where a 10-week community-based Tae-Bo programme produced significant improvements in several fitness and anthropometric outcomes.

Additional Mathunjwa publications provide complementary contextual evidence. The 2023 exercise study in physically inactive college-aged smokers reported improvements in selected lung-function and recovery-heart-rate outcomes, while the rural Tae-Bo programme reported improvements in anthropometric, flexibility, balance, muscular-fitness, and cardiorespiratory measures. These findings illustrate that intervention outcomes are influenced by the target population, programme design, and delivery context, while the evidence remains setting-specific (41).

### 5.2 Implementation, adaptation and scalability

The evidence does not support a simple binary distinction between professional-led HIC interventions and peer-led LMIC interventions. Rather, delivery models appear to be adapted to available workforce, infrastructure, technology, funding, and community preferences. In Massachusetts, Whooten et al. (24) evaluated implementation of the BOKS before-school programme in three schools. The programme enrolled 188 of 230 available places, with 128 children providing evaluation data; mean moderate-to-vigorous activity during sessions was 16.3 min, and the evaluation documented school-level adaptations. In Hong Kong, Lee et al. (32) used educational workshops and a mobile application alongside outdoor exercise facilities, illustrating a technology-supported model in a high-income context. These studies demonstrate that implementation adaptation occurs within both resource-rich and resource-constrained environments.

The additional Mathunjwa literature reinforces these contextual dimensions. Community-centred strategies for T2DM and HIV emphasise culturally relevant, scalable and inclusive approaches in resource-constrained settings, while work on indigenous games highlights the potential contribution of culturally familiar physical activities to NCD prevention in African communities (35, 36). These sources are contextual and narrative rather than pooled quantitative evidence and are therefore used to illustrate implementation and cultural considerations.

### 5.3 Navigating contextual modifiers

Contextual factors influence whether an intervention is acceptable, accessible, and sustainable. The evidence supports attention to gender, culture, safety, health literacy, social support, and the physical environment, but the direction and importance of each factor are context-specific. The Mathunjwa et al. (31) study demonstrates how a structured group intervention can be implemented in a rural South African community despite resource constraints, while the Hong Kong mHealth study illustrates the importance of smartphone access and engagement for technology-supported delivery. These examples caution against treating contextual modifiers as static barriers; they can also be design opportunities when interventions are adapted collaboratively with communities.

### 5.4 Sustainability, evidence strength and policy integration

Sustainability should be considered separately from short-term efficacy. The Hassen et al. (21) synthesis noted that intervention effects can decline at longer follow-up, while implementation studies such as Whooten et al. (24) show that programme maintenance depends on organisational capacity and adaptation. Evidence supporting policy integration is therefore stronger when linked to identified implementation mechanisms rather than assumed from national income status. Across settings, sustainability is likely to depend on financing, workforce capacity, community ownership, monitoring, infrastructure, and alignment with existing health and social systems.

### 5.5 Limitations of the review and evidence base

The principal limitations are the heterogeneity of the available evidence, restriction to English-language sources, reliance on published and institutional literature, uneven representation of LMIC settings, and the relevance-based selection inherent in a narrative review. The review did not use a preregistered systematic-review protocol, formal PRISMA screening, or a uniform risk-of-bias instrument across all source types. Several representative sources are narrative or implementation-focused and therefore do not provide directly comparable effect estimates. The evidence should therefore be interpreted as a contextual synthesis rather than a pooled estimate applicable to all HIC or LMIC settings. In addition, country income classifications are used as contextual descriptors and should not be interpreted as causal explanations of intervention effects.

## 6 Conclusion

The evidence supports community-based physical activity as a relevant component of NCD prevention and management, but the literature does not justify treating HICs and LMICs as internally uniform categories. Quantitative evidence demonstrates improvements in physical activity and related outcomes, while context-specific studies show that delivery models must be adapted to infrastructure, workforce, technology, culture, safety, and community needs. The Layered Socio-Ecological Comparison Framework provides an organising structure for interpreting these differences and should be understood as an author-developed conceptual synthesis rather than a validated standalone theory.

### 6.1 Recommendations for policy and practice

Policy and practice should prioritise context-sensitive implementation rather than the direct transfer of a single intervention model. In LMIC settings, planning should consider affordability, community health workers or peer support, use of existing community spaces, continuity of funding, and integration with primary health systems. In HIC settings, technology-supported and professionally delivered interventions should still address access, equity, adherence, and local preferences. Across settings, community participation, cultural responsiveness, safe environments, appropriate monitoring, and sustainable financing should be incorporated from the outset.

### 6.2 Future research

Further research should strengthen the evidence base in LMIC settings through adequately described implementation and effectiveness studies, longer follow-up, and transparent reporting of contextual factors. Comparative studies should examine which components of digital, peer-led, group-based, and professionally led interventions are transferable across settings and which require local adaptation. Future reviews using systematic-review methodology should pre-register protocols, report PRISMA flow diagrams, apply formal risk-of-bias tools, and consider quantitative synthesis where sufficient homogeneity exists. Long-term sustainability of peer-led models in resource-constrained environments and the transferability of digital health strategies to underserved populations should receive particular attention.

### 6.3 Final remarks

The principal contribution of this review is the comparative organisation of heterogeneous community-based physical activity evidence around structural context, implementation, behavioural mechanisms, contextual modifiers, and outcomes. The framework is intended to generate testable propositions for future research rather than to substitute for established theory or validated implementation frameworks.
